# Silencing of *RhoA *and *RhoC *expression by RNA interference suppresses human colorectal carcinoma growth in vivo

**DOI:** 10.1186/1756-9966-29-123

**Published:** 2010-09-09

**Authors:** Haibo Wang, Gang Zhao, Xiangping Liu, Aihua Sui, Kun Yang, Ruyong Yao, Zongbao Wang, Qiang Shi

**Affiliations:** 1 Department of General Surgery, Affiliated Hospital of Qingdao University Medical College, Qingdao, 266003, China; 2 Department of Anorectum, Affiliated Hospital of Qingdao University Medical College, Qingdao, 266003, China; 3 Central Laboratory of Molecular Biology, Affiliated Hospital of Qingdao University Medical College, Qingdao, 266003, China

## Abstract

**Background:**

*RhoA *and *RhoC *have been proved to be over-expressed in many solid cancers, including colorectal cancer. The reduction of *RhoA *and *RhoC *expression by RNA interference (RNAi) resulted growth inhibition of cancer cells. The present study was to evaluate the effect of silencing of *RhoA *and *RhoC *expression by RNAi on growth of human colorectal carcinoma (CRC) in tumor-bearing nude mice in vivo.

**Methods:**

To establish HCT116 cell transplantable model, the nude mice were subcutaneously inoculated with 1.0 × 10^7 ^HCT116 cells and kept growing till the tumor xenografts reached 5-7 mm in diameter. Then the mice were randomly assigned to three groups(seven mice in each group): (1) normal saline(NS) group, (2)replication-defective recombinant adenovirus carrying the negative control shRNA (Ad-HK) group and (3)replication-defective recombinant adenovirus carrying the 4-tandem linked *RhoA *and *RhoC *shRNAs (Ad-RhoA-RhoC) group. Ad-HK (4 × 10^8 ^pfu, 30 ul/mouse), Ad-RhoA-RhoC (4 × 10^8 ^pfu, 30 ul/mouse) or PBS (30 ul/mouse) was injected intratumorally four times once every other day. The weight and volumes of tumor xenografts were recorded. The levels of *RhoA *and *RhoC *mRNA transcripts and proteins in tumor xenografts were detected by reverse quantitative transcription polymerase chain reaction (QRT-PCR) and immunohistochemical staining respectively. The terminal deoxynucleotidyl transferase-mediated dUTP nick end labeling (TUNEL) assay was used to detect the death of cells.

**Results:**

The xenografts in mice could be seen at 5th day from the implantation of HCT116 cells and all had reached 5-7 mm in size at 9th day. After injection intratumorally, the growth speed of tumor xenografts in Ad-RhoA-RhoC group was significantly delayed compared with those in NS and Ad-HK group(P < 0.05). The results of QRT-PCR showed that mRNA levels of *RhoA *and *RhoC *reduced more in Ad-RhoA-RhoC group than those in NS and Ad-HK group. The relative *RhoA *and *RhoC *mRNA transcripts were decreased to 48% and 43% respectively (P < 0.05). Immunohistochemical analyses of tumor xenograft sections also revealed the decreased *RhoA *and *RhoC *expression in Ad-RhoA-RhoC group. TUNEL assay also showed higher death of tumor xenograft tissue cells in Ad-RhoA-RhoC group.

**Conclusion:**

Recombinant adenovirus mediated *RhoA *and *RhoC *shRNA in tandem linked expression may inhibit the growth of human colorectal tumor xenografts in vivo. These results indicate that *RhoA *and *RhoC *might be potential targets for gene therapy in colorectal cancer.

## Background

Colorectal carcinoma (CRC) is one of the most common cancers and accounts for about 10% of all new cancer cases and cancer deaths in the US in recent two years[1,2]. And the incidence is increasing rapidly in developing countries including China[3]. Despite surgical resection coupled with systemic chemotherapy, about half of newly diagnosed colorectal cancer patients will still die of this disease due to tumor recurrence and metastasis[4]. The initiation, development, local invasion and distal metastasis for tumor are regulated by multiple genes, whose expressions are determined by either internal or external factors. Therefore, elucidation of those factors and the pattern of their expression may help to understand the mechanism of carcinogenesis and metastasis of colorectal carcinoma.

*RhoA *and *RhoC *have been known to be involved in regulating multiple aspects of cell migration, affecting the different components of the cytoskeleton as well as cell-substrate adhesion and possibly matrix remodeling[5,6]. *RhoA *and *RhoC *proteins have implicated them as important factors in promoting the uncontrolled proliferation and invasive and metastatic properties of cancer cells[7], however, it is poorly understood how they are activated in cancer cells. Studies have demonstrated that the over-expression of *RhoA *and *RhoC *in most solid malignancies including colorectal cancer is more frequently than in normal tissue[8-13]. Therefore, specific inhibiting the functions of *RhoA *and *RhoC *is predicted to be of great therapeutic benefits.

Previous studies have shown that interfering the expression of *RhoA *and *RhoC *using small interfering RNA (siRNA) approaches inhibited the proliferation and invasion of some cancer cells[14-17]. Our previous studies have also demonstrated that the over-expression of *RhoA *and *RhoC *occured in colorectal cancer tissues from Chinese patients and *RhoA *and *RhoC *shRNAs in tandem linked expression could markedly inhibit the invasion and migration potentials of colorectal cancer cells[18,19]. In this study, we evaluated the inhibitory efficacy of *RhoA *and *RhoC *shRNAs in tandem linked expression in vivo. Our results showed that the recombinant adenovirus-mediated siRNA inhibited the growth of colorectal cancer cell grafts implanted in nude mice, which suggests that *RhoA *and *RhoC *might serve as potential targets for gene therapy in colorectal cancer and such shRNA-induced in tandem linked RNA interference might be more effective in targeting multiple genes in cancer therapy.

## Methods

### Experimental Animals

Twenty-one athymic nude male BALBC/c mice(4-5 weeks old, 15-18 g), obtained from Beijing Experimental Animal Center, China, were maintained in a specific pathogen free, temperature-controlled isolation conditions, fed with sterilized food and autoclaved water Animal breeding, care and experiment procedures were approved by ethical and humane committee of Affiliated Hospital of Qingdao University Medical College and carried out strictly in accordance with the related ethical regulations.

### Cell Line and Cell Culture

The human colon cancer cell line HCT116 was purchased from China Centre for Type Culture Collection. The cells were grown in McCoy's 5A medium, Modified (Sigma), supplemented with 10% of fetal bovine serum (Hyclone, USA) at 37°C in a humidified atmosphere of 5% CO_2_. The cells were always detached using 0.25% trypsin and 0.02% ethylene diamine tetra acetic acid(EDTA).

### *In vivo *Tumor Xenograft Model

To establish the transplantable model, the human colon cancer cells in logarithm growth phrase were harvested and washed twice with PBS. 1.0 × 10^7 ^cells in 200 uL of PBS with a viability of >95% tested by staining with trypan blue were injected subcutaneously into the right flank of each mouse. All nude mice were observed to generate tumors for up to 9 days after the injection. When tumor nodules reached 5-7 mm in diameter, tumor model was successfully established and mice were randomly assigned to the following 3 groups(seven mice in each group): (1)normal saline(NS) group, (2) Ad-HK group and (3) Ad-RhoA-RhoC group. Ad-HK (4 × 10^8 ^pfu, 30 ul/mouse), Ad-RhoA-RhoC (4 × 10^8 ^pfu, 30 ul/mouse) or PBS (30 ul/mouse) was injected intratumorally at several points four times once every other day, with the accumulated doses of 1.6 × 10^9 ^pfu. The tumor sizes were determined every other day by external measurements with a vernier caliper and calculated the tumor volume and plotted against time [The tumor volume = ab^2^/2, where a and b are the larger and smaller diameter, respectively]. Ten days after the final injection, the tumors were dissected and their weights and volumes were measured. Then, each harvested tumor was divided into two parts, one was used for detecting the mRNA expression of the related genes and the other was used for immunohistochemical analysis as described below.

### Quantitative RT-PCR for RhoA and RhoC in Xenograft Tumors

Total RNA was extracted from -80°C freezed transplanted tumor samples, dissected from nude mice, using Trizol reagent(Invitrogen, USA) and reverse transcripted into cDNA using the PrimeScript RT-PCR kit (TaKaRa Bio Inc., Shiga, Japan), according to the manufacturer's instructions. To assess the *RhoA *and *RhoC *gene expression, we used real-time fluorescence quantitative PCR analysis based on the TaqMan probe method. The probe contains 6-carboxy-fluorescein (FAM) as a fluorescent reporter dye, and 6-carboxytetramethyl-rhodamine (TAMRA) as a quencher for its emission spectrum. The primers, TaqMan probes and PCR parameters were performed same as reported previously by us [18,19].

### Histopathological and Immunohistochemical Study

Half of the implanted tumor tissues which were dissected from nude mice were fixed in 10% formalin, embedded in paraffin and serially cut into 4 um-thick sections for hematoxylin-eosin and immunohistochemical staining. Briefly, tissue sections were baked, deparaffinized and microwaved at 98°C for 10 minutes in citrate buffer (0.01 M citric acid, pH6.0). After blocking the endogenous peroxidase by immersed the sections in 3% H_2_O_2_, the sections were incubated with primary antibodies directing against human *RhoA *(sc-32039, 1:50; Santa Cruz) and *RhoC *(sc-12116, 1:50; Santa Cruz). Expression of *RhoA *or *RhoC *protein in tissue sections was detected with Anti-goat IgG/HRP Detection Kit(PV-6003; Zhongshan Biotechnology Limited Company, Beijing, China). The tissue sections were then counterstained with hematoxylin.

### Terminal Deoxynucleotidyl Transferase-mediated dUTP Nick End-labeling (TUNEL) Assay

Assessment of cell death was performed by TUNEL method using an *in situ *cell death detection kit conjugated with horse-radish peroxidase (POD) (Roche Applied Science, Indianapolis, IN, USA), according to the manufacturer's instructions. Five equal-sized fields in tissue sections were randomly chosen and analyzed under the Leica DMI 4000B(Leica, Germany) light microscope. Density was evaluated in each positive staining field, yielding the density of dead cells (cell death index).

### Statistical Analysis

All data were shown by mean ± SD. Statistical analyses were performed using SPSS statistical software (SPSS Inc., Chicago, Illinois). Differences between two groups were assessed using a *t *test. A *P *value less than 0.05 was considered statistically significant.

## Results

### Ad-RhoA-RhoC-siRNA Inhibits Tumor Development in Nude Mice

**Figure 1:**
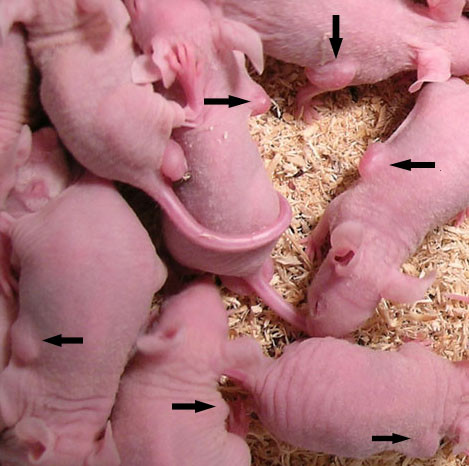
**Tumor-bearing nude mice with 100% of tumor implantation rate**.

**Figure 2:**
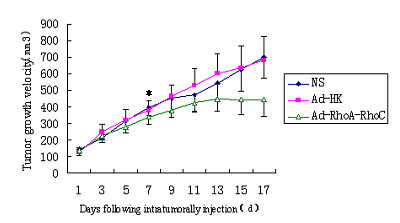
**Growth curve of subcutaneous implanted tumors in nude mice treated with NS, Ad-HK, or Ad-RhoA-RhoC**. Tumor volume is plotted against time elapsed. A significant delay in tumor growth is seen in the group treated with Ad-RhoA-RhoC. Data are presented as means±SD (n = 7). * *P *< .05, from this point onwards.

**Figure 3:**
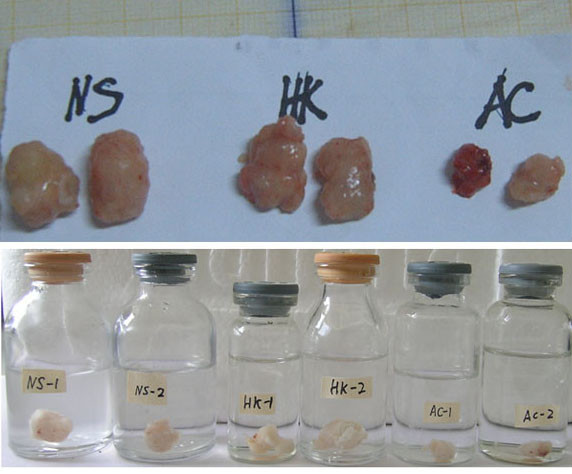
**Comparison of the size of harvested implanted tumors in nude mice treated with NS, Ad-HK, or Ad-RhoA-RhoC**. A: fresh anatomized B: formalin-fixed.

Tumors in the nude mice could be seen at 5th day from the implantation of HCT116 cells and all tumors had reached 5-7 mm in size at 9th day. The successful rate of tumor implantation was 100%(Figure 1). After intratumorally injection, the growth speed of tumors in the three group was quite different. As shown in figure 2, the tumors in NS and Ad-HK group grew rapidly. In contrast, tumors in Ad-RhoA-RhoC group were significantly delayed. The dissected tumors in the NS and Ad-HK group had volumes of (699.62 ± 190.56)mm^3 ^and (678.81 ± 155.39)mm^3^, which were 5.05 ± 0.48-fold and 4.58 ± 0.94-fold larger than the starting volume, whereas in the Ad-RhoA-RhoC group, the tumors had a volume of (441.38 ± 63.03)mm^3^, increased only 2.38 ± 0.56-fold (Figure 3). Tumor growth delay was statistically significant (P < 0.05). In addition, the mean tumor weight in NS, Ad-HK and Ad-RhoA-RhoC group was (0.75 ± 0.22) g, (0.78 ± 0.22) g and (0.36 ± 0.13) g, respectively. These data demonstrated that injection of Ad-RhoA-RhoC was able to slow down the growth of HCT116-derived xenografts.

### Effect of Ad-RhoA-RhoC on Expression of RhoA and RhoC mRNA in Implanted Tumors

PCR product electrophoresis analysis clearly demonstrated a single RhoA band at 158 bp, RhoC band at 136 bp and GAPDH band at 150 bp, which were the expected sizes (figure not shown). Real-time fluorescence quantitative PCR analyses showed the mRNA levels of *RhoA *and *RhoC *were significant decreased in Ad-RhoA-RhoC group compared with the NS group (*P *< 0.05, Table 1). The relative *RhoA *and *RhoC *mRNA expression in Ad-RhoA-RhoC group to the NS group were only about 48% and 43%, respectively. However, there was no significant difference between NS group and Ad-HK group (*P *> 0.05). The results showed that the *RhoA *and *RhoC *genes were specifically silenced in Ad-RhoA-RhoC group.

**Table 1:** The level of *RhoA *and *RhoC *transcripts in implanted tumors in different groups.

Group	*RhoA*		*RhoC*	
	
	ΔΔCT	**Rel. to NS**^**a**^	ΔΔCT	**Rel. to NS**^**a**^
NS	0 ± 0.22	1 (0.86-1.16)	0 ± 0.26	1 (0.84-1.20)
Ad-HK	0.09 ± 0.18	0.94(0.83-1.06)	0.12 ± 0.15	0.92(0.83-1.02)
Ad-RhoA-RhoC	1.05 ± 0.27	0.48(0.40-0.58)	1.23 ± 0.14	0.43(0.39-0.47)

a. Data are expressed as the mean 2^-ΔΔCT ^(range).

### Immunohistochemical Staining for RhoA and RhoC in Xenograft Tumor

**Figure 4:**
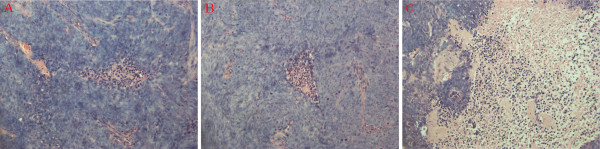
**Tumor tissues in nude mice in different treated groups (HE, ×200) A: NS group; B: Ad-HK group; C: Ad-RhoA-RhoC group**. Tumor cells were intensely stained with hematoxylin and showed smaller sizes. Necrotic regions were mainly eosin stained.

**Figure 5:**
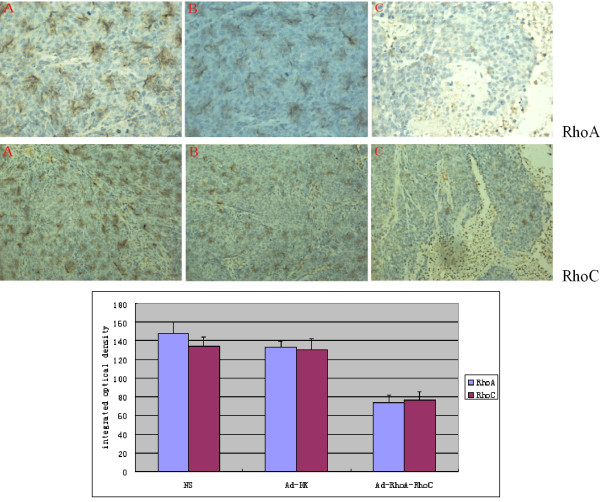
**Immunohistochemistry reaction for *RhoA *and *RhoC *protein in implanted tumor tissues of nude mice in different treated groups (*RhoA*, ×400, *RhoC*, ×200)**. Fig 5 also showed the integrated optical density (IOD) values of the implanted tumor tissues. A: NS group; B: Ad-HK group; C: Ad-RhoA-RhoC group. The positive cells were stained brown, using antibodies to *RhoA *or *RhoC*.

The results of hematoxylin-eosin staining for the pathological changes in tumors were observed under light microscopy (Figure 4). Many necrotic regions were found in the tumors in all the three groups. But in the Ad-RhoA-RhoC group, cancer cells showed intense positive staining with smaller cell sizes and contracted nucleus. Immunohistochemical staining results for *RhoA *and *RhoC *were shown in Figure 5. In Ad-RhoA-RhoC group, the cancer cells of tumor tissues stained very weakly for *RhoA *and *RhoC*, in comparison with NS group and Ad-HK group. Through quantitative data analysis using the Leica Qwin image processing and analysis software (Leica Imaging Solution Lid., Version 3.3.1, Cambridge, UK), the integrated optical density (IOD) values of tumor tissues of NS group, Ad-HK group and Ad-RhoA-RhoC group were 148.02 ± 9.62, 133.44 ± 7.24, 73.51 ± 7.06 for *RhoA *and 134.53 ± 4.51, 130.74 ± 3.78, 76.23 ± 2.17 for *RhoC*, respectively.(Figure 5).

### Death Assay of Cells of Tumor Tissues by TUNEL

**Figure 6:**
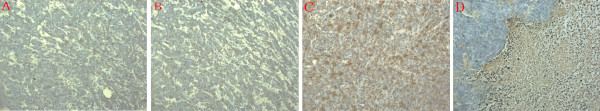
**Cell death in implanted tumor tissues**. Cell death was detected by TUNEL assay in implanted tumors treated with NS(A), Ad-HK(B), or Ad-RhoA-RhoC(C and D). Original magnification, ×200. The nuclei of positive cell were stained brown.

As shown in Figure 6, cancer cells of tumor tissues in Ad-RhoA-RhoC group demonstrated extensive cell death, whereas in NS group and Ad-HK group resulted in less tumor cell death. These results indicate that the induction of cell death by *RhoA-RhoC *siRNA treatment is highly specific.

## Discussion

It has been known that the initiation, development, invasion and metastasis for colorectal carcinoma are controlled by many different genes and various signal transduction pathways and involved in many important biological processes. *RhoA *and *RhoC*, the Rho-related members, have been identified to be involved in diverse signal transduction pathways that control essential cellular functions such as cell growth, cell differentiation, cytoskeletal organization, intracellular vesicle transport and secretion[20]. Despite the high homology of *RhoA *and *RhoC*, *RhoA *has been shown to regulate the activities of multiple transcription factors, most of which are implicated in the cancer progression [21] by modulating cancer cell adhesion, contraction, movement, release of cellular adhesion, degradation of extra-cellular matrix, and invasion into blood or lymph vessels [22,23], while *RhoC *contributes to tumor development, especially to invasion and metastasis of cancer cells [24,25]. But the molecular mechanisms were still unclear.

Previous studies including ours have demonstrated that the overexpression or up-regulation of *RhoA *and *RhoC *in colorectal cancer was significantly higher than those in the corresponding paratumor and normal tissues, suggesting the involvement of these two genes in the onset, development and disease progression. of colorectal carcinoma [11,12,18,26]. Moreover, some reports showed that down-regulating the expression of *RhoA *and *RhoC *using small interfering RNA (siRNA) approaches may inhibit the proliferation and invasiveness of cancer cells [14-17,19,27]. Therefore, specific inhibiting the abnormal expression of *RhoA *and *RhoC *may be an effective strategy for CRC therapy.

Now, RNA interference has become widely used *in vivo *knockdown of genes in cancer therapy. However, safe, feasible and effective delivery methods in vivo are still of critical importance[28]. Viral vectors do possess significant advantages in cancer therapy in vivo and gene therapy with intratumorally injected recombinant adenoviral vectors mediating sequence-specific gene silence offers the potential to restrict therapeutic gene expression in the tumor. Thus, the use of RNAi in a stable viral vector system, such as the adenovirus, is a highly desirable strategy for stable gene knockdown in anticancer gene therapy[29-31].

Our previous *in vitro *data have demonstrated that our recombinant adenovirus mediated *RhoA *and *RhoC *shRNA in tandem linked expression successfully inhibits the expression of *RhoA *and *RhoC *in CRC cell line HCT116 and proliferation of CRC cells. On the basis of *in vitro *results, the present study was aimed to determine whether the recombinant adenovirus mediated 4-tandem linked shRNA construct targeting *RhoA *and *RhoC *genes may inhibit the growth of human colorectal cancer cell graft implanted in nude mice *in vivo*. Our results indicated that the growth speed of the implanted tumors in NS, Ad-HK and Ad-RhoA-RhoC groups was quite different after intratumoral injection of NS, Ad-HK and Ad-RhoA-RhoC respectively. The tumor weight and the tumor volume were significantly declined in Ad-RhoA-RhoC group. RT-PCR and immunohistochemistry results showed that the mRNA and protein expressions of *RhoA *and *RhoC *were markedly decreased in Ad-RhoA-RhoC group. The TUNEL study also disclosed that increased dead cells in this group compared with those in NS and Ad-HK group. These results showed that the recombinant adenovirus mediated *RhoA *and *RhoC *shRNA in tandem linked expression could inhibit the growth of tumors in CRC-bearing nude mice.

To our knowledge, this is the first study that 4-tandem linked shRNA construct targeting *RhoA *and *RhoC *genes can inhibit the growth of colorectal tumors in *vitro *and in *vivo*. *RhoA *and *RhoC *gene may be promising molecular targets for colorectal cancer gene therapy. Although, there are three mice in NS and Ad-HK group died one or two days before the harvest day in our study, we think this is irrelative to the adenovirus application but owing to their large tumors or cachexia. All the data we observed about the adenovirus application shows no any serious side effects(data not shown), which means that adenoviral vector-based delivery of in tandem linked shRNAs is a safe and efficient therapeutic approach. There weren't any differences such as body weight, implanted tumor weight, etc. between NS and Ad-HK group. However, we have kept doing research work on comparing the inhibitory effects of multiple shRNAs expression vectors with single shRNA expression vector. And further research work should be done to examine the downstream effectors of RhoA and RhoC; such as ROCK-I and ROCK-II, being most associated with metastasis and progress in cancer, which will be benefit for exploring the possible molecular mechanisms of RhoA and RhoC in tumor inhibition.

## Competing interests

The authors declare that they have no competing interests.

## Authors' contributions

WHB and LXP designed the research; YK and SAH carried out the molecular genetic studies; WZB and SQ participated in the nude mice studies; ZG and YRY discussed the results and analyzed data; WHB and LXP wrote the paper. All authors read and approved the final manuscript.
